# Development of a Practical Nomogram for Depression Risk Stratification in Older Adults With Hypertension and Diabetes: Retrospective Analysis of Data From the China Health and Retirement Longitudinal Study

**DOI:** 10.2196/81529

**Published:** 2026-03-12

**Authors:** Ting Peng, Ying Zhang, Rujia Miao, Jiangang Wang

**Affiliations:** 1 Health Management Center, The Third Xiangya Hospital, Central South University, 138 Tongzipo Road, Yuelu District, Changsha, Hunan, 410013, China, 86 18692269664

**Keywords:** depression risk prediction, hypertension, diabetes, activities of daily living, nomogram

## Abstract

**Background:**

Depression affects over 40% of middle-aged and older Chinese adults living with both hypertension and diabetes, amplifying cardiovascular risk, functional decline, and mortality. Existing screening instruments—such as the 10-item Center for Epidemiologic Studies Depression Scale—focus narrowly on mood symptoms and are rarely feasible in busy primary care consultations. They also omit routine functional, cognitive, and social data that may jointly drive depressive states in cardiometabolic populations.

**Objective:**

This study aimed to develop and validate a concise, clinically actionable nomogram that quantifies individual depression risk using readily available information in Chinese adults aged ≥45 years who have diagnosed hypertension and type 2 diabetes.

**Methods:**

We analyzed anonymized wave 5 China Health and Retirement Longitudinal Study data collected between July 2020 and August 2020. Of 1504 eligible participants, 635 (42.2%) met the Center for Epidemiologic Studies Depression Scale cutoff score of >10 for probable depression. A total of 42 candidate predictors spanning demographics, laboratory values, comorbidities, functional status, and socioenvironmental factors were screened. Least absolute shrinkage and selection operator regression with 10-fold cross-validation identified the most parsimonious set. A multivariable logistic model was built on a 70% training set (n=1052) and evaluated on a 30% testing set (n=452). Performance was assessed using the area under the receiver operating characteristic curve (AUC), calibration plots, decision curve analysis, and Shapley additive explanations for interpretability. Multiple imputation was used to handle <20% missingness.

**Results:**

Nine nonredundant predictors entered the final nomogram: activity of daily living score, memory impairment, number of pain sites, sleep duration, life satisfaction score, self-rated health score, social activity engagement score, retirement status, and memory test score. The model achieved excellent discrimination (training AUC=0.819; testing AUC=0.825) and calibration (mean absolute error ≤0.018). Decision curves demonstrated positive net clinical benefit across clinically relevant threshold probabilities. Shapley additive explanations analysis revealed a 3-fold increase in depression odds per 1-point increase in activity of daily living score, whereas retirement conferred substantial protection (prevalence of depression: 103/635, 16.2% in the retired group vs 269/869, 31.0% in the nonretired group; *P*<.001), mediated by greater social participation.

**Conclusions:**

The 9-item nomogram enables <3-minute depression risk stratification in resource-limited primary care settings for adults with hypertension and diabetes. Functional decline, affective-cognitive burden, and socioeconomic disengagement constitute the dominant causal pathway. Prospective trials should examine whether interventions targeting postretirement social engagement and functional rehabilitation can reduce incident depression in this high-risk population.

## Introduction

Depression imposes an additional burden on people living with chronic conditions, especially among middle-aged and older adults who are trying to manage both high blood pressure and diabetes [1]. These physical illnesses often appear together with depressive symptoms, making treatment harder and speeding up the loss of daily function [2]. Studies have shown that, in this group, the rate of depression can reach more than 40% in some samples, and it is linked to poorer treatment adherence, higher heart-related risks, and greater chances of early death [3,4]. Even though depression is clearly important, it is still missed in many primary care visits [5]. The screening tools we have are reliable on paper, but they are hard to fit into busy clinics because of limited time and other practical barriers. The 10-item version of the Center for Epidemiologic Studies Depression Scale (CESD-10), widely used in Chinese-speaking populations, is a good example: when used alone, it cannot draw on the wider set of clinical, behavioral, and social clues that would help physicians spot depression earlier [6,7].

Most earlier models for predicting depression were built for people receiving psychiatric care or used general risk factors, so they do not work well for people who also have long-term illnesses [8,9]. A key weakness is that these models rarely include measures of daily function, such as the ability to bathe, dress, cook, and handle money, together with behavioral and social factors [10]. This omission matters because poor physical function can be both an early sign of depression and a result of uncontrolled blood pressure or blood sugar, making it hard to know which came first [11]. For these reasons, we set out to create a simple, practical tool to estimate depression risk in middle-aged and older adults with hypertension and diabetes. Using data from the China Health and Retirement Longitudinal Study (CHARLS), we examined 42 possible predictors covering medical, psychological, and social areas. Using a logistic regression model improved through machine learning and checked through several validation steps, we carried out 3 tasks: choosing the smallest set of predictors that still separates high-risk from low-risk individuals; turning these predictors into a clear, easy-to-use score that can be applied in busy, low-resource clinics; and showing, with numbers, how much heart- and metabolism-related functional problems act as a link between illness and depression. Rather than focusing only on symptoms, our approach places depression risk within the patient’s overall health picture. The resulting model can guide clinicians and also point to potentially modifiable risk factors.

## Methods

### Ethical Considerations

This study used anonymized data from the fifth wave of the CHARLS. The project was approved by the Peking University Biomedical Ethics Committee in June 2008 (approval IRB00001052–11015). All personal identifiers were removed before our analysis. Patients and the public were not involved in the design, conduct, reporting, or dissemination of this research. All participants provided written informed consent, and the study followed the ethical principles set out in the Declaration of Helsinki. No financial or material compensation was offered to study participants.

### Study Population

We carried out a retrospective study using data from the fifth wave of the CHARLS collected between July 2020 and August 2020. Participants had to be aged at least 45 years and have both hypertension and diabetes. We excluded anyone who was missing key details such as CESD-10 score, diagnosis of hypertension or diabetes, or age. After these exclusions, 1504 people were left for the analysis. Depression status was based on the CESD-10, a brief questionnaire that mixes positively and negatively worded statements. Each item is scored from 0 to 3, yielding total scores from 0 to 30; higher scores mean more severe depressive symptoms [12,13]. In Chinese older adults, a score above 10 is generally taken to indicate possible depression, and this cutoff has been shown to work well in previous studies and in latent profile analyses [13,14]. Using this cutoff, of the 1504 participants, we labeled 635 (42.2%) as the depression group and 869 (57.8%) as the nondepression group.

### Candidate Predictor Variables

Guided by earlier studies and clinical practice, we chose 42 factors that might relate to depression risk [15-17]. These factors cover basic personal details, overall health, daily habits, blood test results, and how well people manage daily tasks. The full list is as follows: sex, age, marital status, smoking status, drinking status, cancer, lung disease, heart disease, stroke, mental illness, arthritis, high blood fat, liver disease, kidney disease, stomach disease, asthma, memory problems, hip fracture, number of painful body sites, exercise level, sleep hours, social activity engagement, whether the patient was retired, medical insurance, pension insurance, years of schooling, household size, number of living children, household income, recent physician visits, memory test score, activity of daily living (ADL) score, instrumental ADL (IADL) score, self-rated health, and satisfaction with life. Regarding “social activity engagement,” it was categorized into the following eight types: (1) interacted with friends (visiting friends or socializing with friends); (2) played mahjong, played chess, played cards, or went to community clubs; (3) provided help to family members, friends, or neighbors who do not live with the participant and who did not pay them for the help; (4) went to a sports, social, or other type of club; (5) took part in community-related organization activities; (6) did voluntary or charity work; (7) attended an educational or training course; and (8) other.

Participants completed the questionnaire based on their actual situation: participation in 1 type of the above activities was recorded as “1,” participation in 2 types was recorded as “2,” and a higher count indicated a higher level of social activity engagement of the participant. For “life satisfaction,” the CHARLS survey asked respondents about their overall satisfaction with their lives, with 5 response options: “extremely satisfied,” “very satisfied,” “relatively satisfied,” “not very satisfied,” and “not satisfied at all.” For the convenience of statistical analysis, we assigned numerical values to these levels: “1” represented “not satisfied at all,” “5” represented “extremely satisfied,” and so on, with each level corresponding to a specific number.

### Model Construction Methods

We used least absolute shrinkage and selection operator (LASSO) regression to pick out the most useful risk factors while keeping the list short. Ten-fold cross-validation helped us find the best penalty value (λ). We then split the data into a training set (1052 people) and a testing set (452 people) in a 7:3 ratio. The training set was used to find the key factors linked to depression. These factors were entered into a standard logistic regression, and we drew a simple chart (nomogram) to show how each one affected the risk of depression. To keep the model easy to use, we removed predictors with little impact, reran the logistic regression with the remaining ones, and built the final nomogram.

### Model Evaluation Methods

To check how well the model performed, we used receiver operating characteristic curves and the area under these curves (AUC) to measure overall ability to separate people with and without depression, calibration plots to check whether the predicted chances matched what really happened, and decision curve analysis to weigh the benefits of true positive predictions against the harms of false ones, giving a clear picture of both accuracy and clinical value.

### Model Interpretation Methods

To make the model easier to understand, we used Shapley additive explanations (SHAP) values to show how much each factor mattered. We drew 4 simple charts (an overall importance plot, a swarm plot, a waterfall plot, and a force plot) that displayed how every factor pushed the risk of depression up or down. These pictures help readers see which factors carry the most weight and how they work together in the model.

### Data Processing Methods

All statistical analyses and figure generation were performed using R (R Foundation for Statistical Computing). When less than 20% of the data were missing, we used the *mice* package to run 5 rounds of imputation and kept the set that best matched the overall pattern. We report counts and percentages for categorical variables and means with SDs for continuous variables. We compared groups using 2-tailed *t* tests, chi-square tests, or nonparametric tests as needed; the latter were chosen when the data did not follow the rules for parametric tests. A *P* value <.05 was considered statistically significant.

## Results

### Flowchart

**Figure 1.**
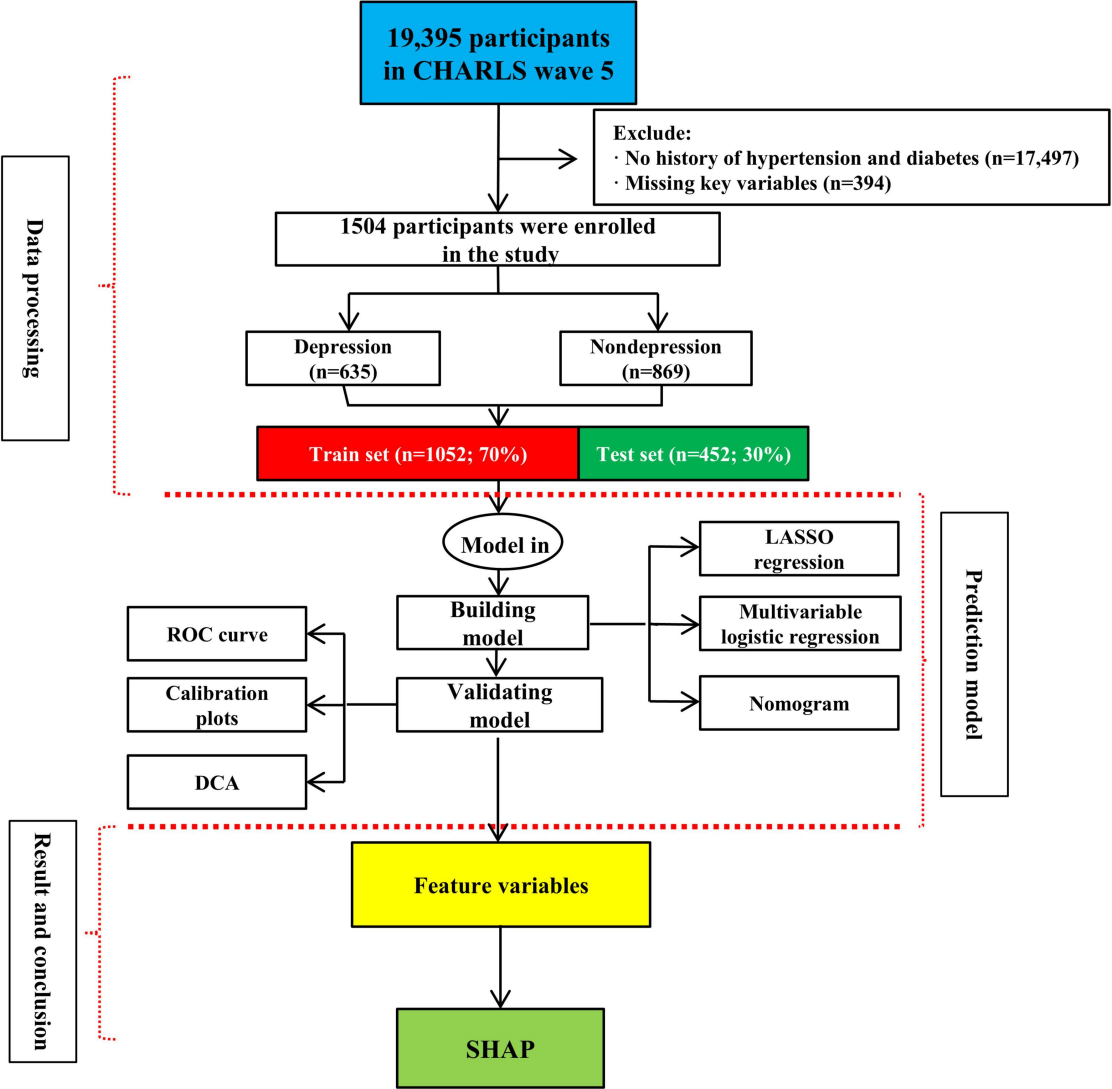
Study flowchart. Analysis of China Health and Retirement Longitudinal Study (CHARLS) wave 5 participants with hypertension and diabetes comorbidity (aged ≥45 years). DCA: decision curve analysis; LASSO: least absolute shrinkage and selection operator; ROC: receiver operating characteristic; SHAP: Shapley additive explanations.

The study flowchart is presented in Figure 1.

### Baseline Characteristics

We included 1504 adults with an average age of 65.99 (SD 8.28) years and 64.93 (SD 8.72) years for those with and without depression, respectively; 678 (45.1%) were men, and 826 (54.9%) were women. Of these 1504 participants, 635 (42.2%) were classed as having possible depression, and 869 (57.8%) were classed as nondepressed. Table 1 shows that the 2 groups differed on many key points. Those with possible depression were more often women, widowed or separated, and less active and reported more illnesses, such as lung disease, heart disease, stroke, arthritis, mental illness, liver disease, kidney disease, stomach disease, asthma, hip fracture, and memory problems. They also had more pain sites; poorer sleep; lower income; fewer years of schooling; smaller households; more physician visits; fewer social activities; and lower scores on memory, daily activities, self-rated health, and life satisfaction (*P*<.05 in all cases).

**Table 1.** Baseline characteristics of study participants by depression status. *P* values were calculated using *t* tests, chi-square tests, or nonparametric tests as appropriate. A *P* value of <.05 was considered statistically significant.

Characteristic	Nondepression (n=869)	Depression (n=635)	*P* value
Medical insurance, n (%)	834 (96.0)	608 (95.7)	.93
Pension insurance, n (%)	765 (88.0)	543 (85.5)	.17
Exercise, n (%)	793 (91.3)	548 (86.3)	.003
Retired, n (%)	269 (31.0)	103 (16.2)	<.001
Sex (male), n (%)	463 (53.3)	215 (33.9)	<.001
Married, n (%)	746 (85.8)	504 (79.4)	.001
Hip fracture, n (%)	9 (1.0)	13 (2.0)	.16
Cancer, n (%)	30 (3.5)	24 (3.8)	.84
Lung disease, n (%)	120 (13.8)	156 (24.6)	<.001
Heart disease, n (%)	326 (37.5)	339 (53.4)	<.001
Stroke, n (%)	98 (11.3)	136 (21.4)	<.001
Psychiatric disease, n (%)	25 (2.9)	58 (9.1)	<.001
Arthritis, n (%)	324 (37.3)	389 (61.3)	<.001
Dyslipidemia, n (%)	572 (65.8)	457 (72.0)	.01
Liver disease, n (%)	90 (10.4)	107 (16.9)	<.001
Kidney disease, n (%)	144 (16.6)	174 (27.4)	<.001
Stomach disease, n (%)	261 (30.0)	265 (41.7)	<.001
Asthma, n (%)	55 (6.3)	85 (13.4)	<.001
Memory problems, n (%)	47 (5.4)	97 (15.3)	<.001
Vigorous physical activity, n (%)	237 (27.3)	193 (30.4)	.21
Moderate physical activity, n (%)	474 (54.5)	319 (50.2)	.11
Light physical activity, n (%)	727 (83.7)	482 (75.9)	<.001
Drinking, n (%)	318 (36.6)	142 (22.4)	<.001
Smoking, n (%)	203 (23.4)	95 (15.0)	<.001
Hospitalization in the previous year, n (%)	241 (27.7)	266 (41.9)	<.001
Recent physician visits, n (%)	227 (26.1)	220 (34.6)	<.001
Total income, mean (SD)	¥54,055.20 (US $7858.73; ¥79,512.32 [US $10,687.50])	¥32,470.65 (US $4720.71; ¥88,119.77 [US $12,811.20])	<.001
Family size (number of individuals), mean (SD)	2.88 (1.56)	2.58 (1.40)	<.001
Number of healthy children, mean (SD)	2.37 (1.25)	2.69 (1.21)	<.001
Financial support from children to parents, mean (SD)	¥6984.23 (US $1015.40; ¥15,871.02 [US $2307.39])	¥5448.44 (US $792.12; ¥8541.60 [US $1241.81])	.03
Financial support from parents to children, mean (SD)	¥9176.07 (US $1334.05; ¥49,285.04 [US $7165.26])	¥4270.93 (US $620.93; ¥21,830.53 [US $3173.81])	.02
Self-rated health score (1-5), mean (SD)	2.82 (0.90)	2.18 (0.89)	<.001
Life satisfaction score (1-5), mean (SD)	3.41 (0.66)	2.87 (0.87)	<.001
Number of pain sites, mean (SD)	2.11 (3.01)	4.53 (4.35)	<.001
Sleep duration (h), mean (SD)	6.21 (1.68)	5.27 (2.06)	<.001
Social activity engagement (number of activities participated in), mean (SD)	0.94 (1.14)	0.65 (0.90)	<.001
Age (y), mean (SD)	64.93 (8.72)	65.99 (8.28)	.02
Educational level^a^, mean (SD)	2.26 (1.11)	1.85 (1.02)	<.001
Total MET^b^ (min), mean (SD)	4627.13 (4936.56)	4832.40 (5150.61)	.43
Memory score (0-11), mean (SD)	4.51 (1.74)	3.61 (1.84)	<.001
ADL^c^ score (0-6), mean (SD)	0.34 (0.85)	1.20 (1.60)	<.001
IADL^d^ score (0-5), mean (SD)	0.32 (0.84)	1.18 (1.50)	<.001

a Educational level was scored as follows: 1=illiterate; 2=below junior high school; 3=junior high school and above, below university; 4=university and above.

b MET: metabolic equivalent of task.

c ADL: activity of daily living.

d IADL: instrumental ADL.

### Results of Model Construction

**Figure 2.**
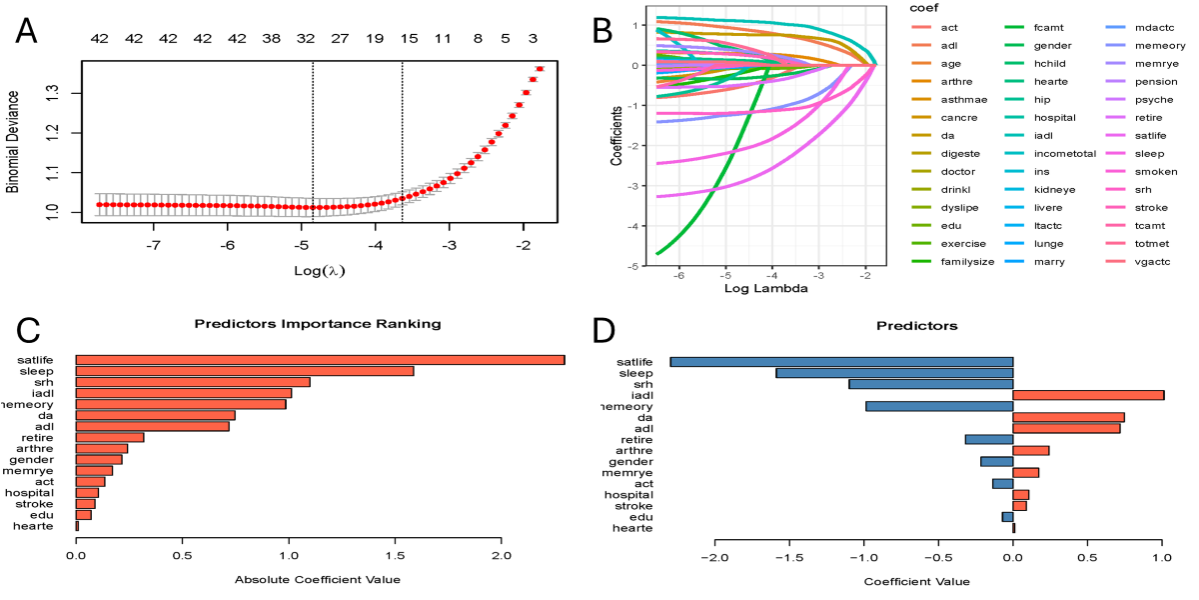
Predictor selection using least absolute shrinkage and selection operator (LASSO) regression. (A) Cross-validation plot showing the relationship between the regularization parameter (λ) and model deviance. The vertical dashed line indicates the optimal λ value (minimum λ+1 SE). (B) LASSO coefficient path plot showing how variable coefficients shrink toward 0 as λ increases. (C) Ranking of the 16 selected predictors based on their standardized regression coefficients. (D) Coefficient direction plot indicating risk (red=positive coefficient) and protective (blue=negative coefficient) factors for depression. Act: social activity engagement; ADL: activity of daily living; DA: number of pain sites; IADL: instrumental ADL; memrye: memory problems; satlife: life satisfaction score; SRH: self-rated health score.

As the regularization strength (λ) increases, the model error initially decreases and then increases. We chose the simplest model within 1 SE of the minimum error. This left 16 variables whose coefficients stayed above 0 (Figure 2A). Each line in the plot shows one variable’s coefficient; as λ grows (moving right), many lines drop to 0 and are removed. In the end, 16 factors remained: life satisfaction score, sleep hours, self-rated health score, IADL score, memory score, pain sites, ADL score, retirement status, arthritis, sex, memory problems, social activity engagement type, recent physician visits, stroke, years of schooling, and heart disease, listed from most to least important (Figure 2C). Figure 2D shows the direction of each factor: red bars mean higher risk, and blue bars mean lower risk.

**Figure 3.**
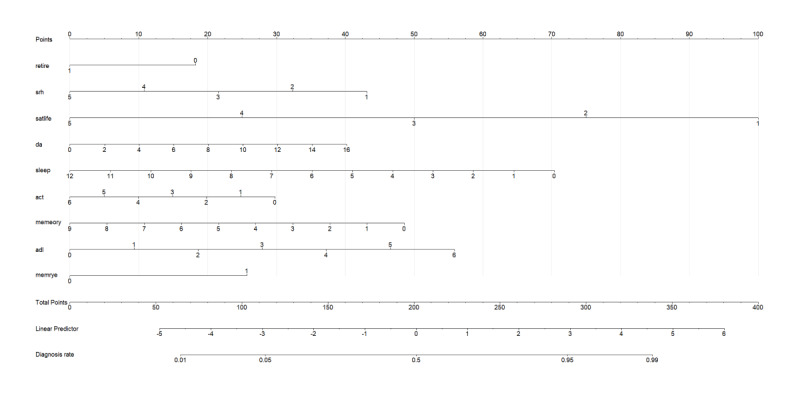
Nomogram for predicting depression risk. The nomogram was constructed using 9 predictors selected from multivariate logistic regression. Each predictor is assigned a point value based on its regression coefficient. The total score corresponds to the predicted probability of depression in the bottom scale. Higher total scores indicate a higher risk of depression. Act: social activity engagement; ADL: activity of daily living; DA: number of pain sites; memrye: memory problems; satlife: life satisfaction score; SRH: self-rated health score.

We took the 16 variables and ran them together in a new regression model and then drew a simple point chart (nomogram). The chart showed which factors mattered the most so that we could drop the weaker ones. After this step, 9 factors remained: retirement status, self-rated health score, life satisfaction score, number of pain sites, sleep hours, social activity engagement type, memory score, ADL score, and memory-related disease. We put these 9 factors into a final regression and built the last nomogram (Figure 3). On the chart, each line shows how many points a person obtains for that factor; the bottom scale turns the total points into the chance of depression. Higher total points mean higher risk.

### Results of Model Evaluation

**Figure 4.**
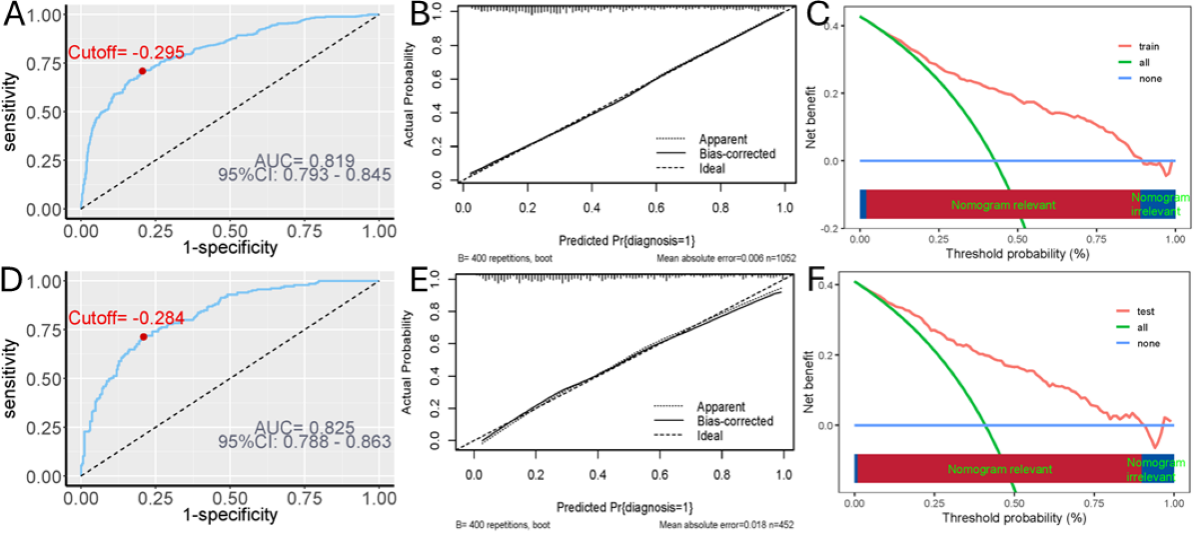
Evaluation of the predictive model. (A) Receiver operating characteristic (ROC) curve and area under the curve (AUC) value in the training set (AUC=0.819). (B) Calibration plot in the training set (mean absolute error=0.006). (C) Decision curve analysis (DCA) in the training set. (D) ROC curve and AUC value in the testing set (AUC=0.825). (E) Calibration plot in the testing set (mean absolute error=0.018). (F) DCA in the testing set. The model demonstrates strong discriminative ability, good calibration, and high clinical utility in both datasets.

We tested how well the model separated people with and without depression using the AUC. In the training data (Figure 4A), the AUC was 0.819, and in the testing data (Figure 4D), it was 0.825, showing good discrimination. The predicted probabilities matched the real outcomes closely in both sets; the average error was only 0.006 in training (Figure 4B) and 0.018 in testing (Figure 4E). Decision curve analysis (Figures 4C and 4F) also showed clear clinical benefit: the model yielded much larger net benefits than the “treat all” or “treat none” approaches. Overall, the nomogram was accurate and clinically useful.

### Results of Model Interpretation

**Figure 5.**
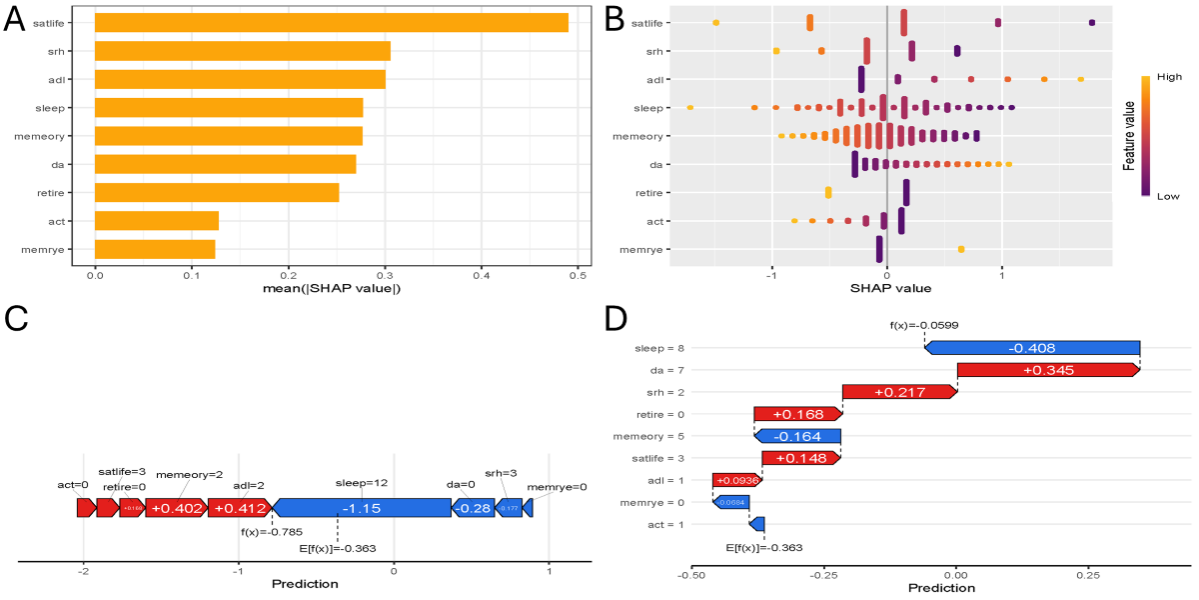
Shapley additive explanations (SHAP) analysis of the final model. (A) Global SHAP importance plot showing the average impact of each feature on model predictions. (B) SHAP swarm plot illustrating the distribution of SHAP values for each feature across all participants (red=increased risk; blue=decreased risk). (C) Waterfall plot for a randomly selected individual showing the contribution of each feature to the final prediction. (D) Force plot for another individual, highlighting the cumulative effect of features on the predicted probability of depression. Positive SHAP values (red) increase the risk of depression, whereas negative values (blue) decrease it. Act: social activity engagement; ADL: activity of daily living; DA: number of pain sites; memrye: memory problems; satlife: life satisfaction score; SRH: self-rated health score.

We used SHAP values to look at the 9 main variables. Global and swarm plots (Figure 5A-B) show how each variable affects the entire sample. ADL score, memory problems, and number of pain sites pushed the risk upward, whereas retirement status, social activity engagement type, sleep hours, life satisfaction score, self-rated health score, and memory score pushed it downward. To see how these factors worked for individual people, we picked 2 participants at random and used waterfall and force plots (Figure 5C-D) to show each factor’s exact contribution.

## Discussion

### Principal Findings

This study establishes a clinically operable prediction model for depression risk among middle-aged and older patients with hypertension and diabetes, leveraging multidimensional predictors to achieve robust discrimination (AUC=0.819‐0.825) and calibration (mean absolute error≤0.018). Nine core variables emerged as significant predictors in the final nomogram: ADL score, memory disorders, number of pain sites, sleep duration, life satisfaction score, self-rated health score, social activity engagement, retirement status, and memory score. The centrality of functional impairment as a mechanistic pathway is underscored by the ADL score’s dominant predictive role [18,19], where individuals with depression exhibited markedly elevated scores (mean 1.20, SD 1.60 vs mean 0.34, SD 0.85; *P*<.001). Each unit increase in ADL score corresponded to a 3-fold increase in depression risk, particularly at critical thresholds beyond ADL score ≥2. Retirement status provided substantial protection against depression [19,20], with prevalence rates nearly halved among retirees (103/635, 16.2%) compared to nonretirees (269/869, 31.0%; *P*<.001) mediated by significantly greater social participation (mean activity score 0.94, SD 1.14 vs mean 0.65, SD 0.90; *P*<.001). SHAP value distributions (Figure 5A-B) corroborated a triadic pathogenesis framework. First, functional autonomy erosion (ADL and IADL decline interacting with multisite pain) imposes physical constraints that amplify helplessness [21,22]. Second, affective-cognitive burden manifests through memory disorders (there were memory disorders in 97/635, 15.3% vs 47/869, 5.4% of participants with and without depression, respectively; *P*<.001), and reduced life satisfaction (mean score 2.87, SD 0.87 vs mean score 3.41, SD 0.66 among participants with and without depression; *P*<.001) depletes emotional reserves [23]. Third, socioeconomic disengagement was evidenced by significantly lower household incomes among participants with depression (mean ¥32,470.65 [US $4720.71], SD ¥88,119.77 [US $12,811.20] vs mean ¥54,055.20 [US $7858.73], SD ¥79,512.32 [US $10,687.50], respectively; *P*<.001), constraining access to health-promoting resources [24,25]. The nonlinear inflection point observed in the ADL-depression relationship indicates that moderate functional decline triggers disproportionately severe psychological sequelae, offering an identifiable intercept for targeted interventions [26].

These findings advance beyond prior depression prediction paradigms in several critical dimensions. Traditional screening instruments reliant on mood-based symptomatology such as the Patient Health Questionnaire series demonstrate limited applicability in cardiometabolic populations due to diagnostic circularity: they cannot resolve whether fatigue or anhedonia stems from depression vs underlying chronic disease pathology [27,28]. Our model effectively dissociates these confounds by operationalizing ADLs as an independent construct validated through SHAP’s feature dissociation capability. For instance, while arthritis prevalence was markedly higher among participants with depression (389/635, 61.3% vs 324/869, 37.3%; *P*<.001), LASSO selected number of pain sites rather than arthritis diagnosis as the principal pain predictor due to its ability to quantify functional impact severity [29]. When benchmarked against models with similar AUC values to those of the model evaluated in this study, such as the depressive symptom predictor by Zheng et al [30,31], our algorithm demonstrates superior translational efficiency by using information already captured in routine chronic disease management ADL documentation during nursing assessments, medication review for memory issues, and social history documenting retirement status. This obviates the need for additional screening instruments, reducing assessment time to under 3 minutes. Moreover, we identify novel modifiable pathways absent in prior frameworks. The protective effect of retirement when linked to sustained social activity engagement reveals life transitions as windows for resilience-building interventions [32]. Enhancing life satisfaction emerged as another actionable leverage point given its strong negative association with depression (β=−0.63 via LASSO), suggesting that psychosocial enrichment may disrupt pathogenic cascades more effectively than purely biomedical approaches [33].

Methodologically, the integration of LASSO regression with SHAP value interpretation constitutes a primary strength. LASSO refined the predictor pool from 42 candidates to 9 nonredundant variables, efficiently eliminating noncontributory markers such as cancer or lung disease. SHAP analysis then transformed statistical coefficients—for example, the negative β value for retirement (−0.89)—into clinically interpretable narratives. At an individual level (Figure 5D), retirees maintaining active social roles exhibited an 18-fold depression risk reduction compared to their socially isolated counterparts. The CHARLS cohort’s population representativeness further enhances generalizability to China’s aging cardiometabolic patient demographic. Four principal limitations warrant acknowledgment. Due to the cross-sectional nature of wave 5 CHARLS data, causal precedence between functional decline and depression could not be definitively established. However, the biological gradient observed in ADL risk proportionality (higher scores predicting depression probability stepwise) lends credibility to functional deterioration as a driver rather than a secondary consequence [34,35]. Second, depression ascertainment using a CESD-10 score >10 may overlook subsyndromal presentations, potentially underestimating risk in highly functional individuals [36]. Third, unmeasured variables influencing both pathways, particularly medication adherence patterns or pharmacodynamic interactions between psychotropics and antihypertensives, were unaccounted for. Finally, sociocultural proxies unique to Chinese aging populations’ children’s financial support demonstrating borderline significance (*P*=.02-.03) were excluded from the final model despite their potential clinical relevance [37]. Future iterations should address this through culturally adapted predictor sets.

This study was conducted based on statistical data from the CHARLS database. Therefore, the results of this study have limitations in terms of applicability to young and middle-aged populations, as well as populations from different regions and ethnic groups. In future research, we may consider combining data from public databases in the Americas, Europe, and other regions to conduct joint modeling, thereby improving the generalization performance of the model. These findings suggest priority areas for future research and clinical application. Longitudinal analyses using multiwave CHARLS data should test the proposed retirement–social engagement–protection pathway, examining whether weekly social activity sustains resilience to depressive symptoms. A 3-tier risk stratification protocol based on nomogram scores may guide care: individuals scoring ≤150 can continue annual screening; those scoring 150 to 250 should receive functional preservation strategies, including task-oriented exercise and cognitive behavioral approaches to improve ADLs, sleep, and social engagement; and those scoring ≥250 would require multidisciplinary management due to high comorbidity. Embedding this algorithm into electronic health records within China’s National Essential Public Health Service Package could enable automated alerts when thresholds are met. Pilot programs should focus on communities where the baseline ADL score is ≥1.5; in this study, 51.2% (325/635) of participants with depression were in this category. Randomized trials are needed to evaluate mediator-targeted interventions, including combined ADL and pain management, structured postretirement social role adaptation, and hemoglobin A_1c_–stratified memory screening in diabetes. Targeting domain-specific vulnerabilities rather than symptoms alone may reduce depression incidence while optimizing primary care resources.

### Conclusions

We developed and validated a depression risk model for middle-aged and older adults with hypertension and diabetes. The nomogram, integrating 9 routinely collected predictors, including ADL score, memory impairment, and pain burden, showed strong discrimination and calibration. Depression risk rose 3-fold for each unit increase in ADL score, whereas retirement was protective. Functional impairment, emotional cognitive burden, and socioeconomic disadvantage formed the dominant risk pathway. LASSO-SHAP integration streamlined predictor selection, enabling screening in under 3 minutes. Prospective trials should test interventions targeting postretirement social engagement and functional rehabilitation, alongside implementing a 3-tier risk stratification protocol in primary care. Targeting these domain-specific risks could reduce depression incidence.

## Abbreviations

ADL: activity of daily living
AUC: area under the receiver operating characteristic curve
CESD-10: 10-item Center for Epidemiologic Studies Depression Scale
CHARLS: China Health and Retirement Longitudinal Study
IADL: instrumental activity of daily living
LASSO: least absolute shrinkage and selection operator
SHAP: Shapley additive explanations
